# Comparative assessment of changes in pharyngeal airway space in cases of obstructive sleep apnoea with a customized mandibular repositioning appliance - a clinical study

**DOI:** 10.5935/1984-0063.20200072

**Published:** 2021

**Authors:** Rezeen Aziz, Sanju Somaiah, Anmol S Kalha, Goutham Reddy, Sunil Muddaiah, Balakrishna Shetty

**Affiliations:** Coorg Institute of Dental Sciences, Orthodontics and Dentofacial Orthopedics - Virajpet - Karnataka - India.

**Keywords:** Sleep Apnea, Obstructive, Snoring, Polysomnography, Sleep Apnea Syndromes

## Abstract

**Objectives:**

The present study aims at evaluating the effects of a customized mandibular repositioning appliance on the pharyngeal airway, nocturnal sleep patterns, daytime discomfort and occlusal changes in established cases of adult obstructive sleep apnoea.

**Material and Methods:**

Ten consecutive patients with a complaint of snoring and disturbed sleep were included in the study. The primary diagnosis was established by the Epworth sleepiness scale, clinical examination, history and subsequently the diagnosis was substantiated through assessment of the pharyngeal airway space on a lateral cephalogram and polysomnography. A customized mandibular repositioning appliance was used to advance the mandible sequentially every 6 months, using 4 sets of the appliance. Pre and post-treatment evaluations were performed to establish, effects and changes in the outcome of obstructive sleep apnoea.

**Results:**

The study revealed significant increase in the mean pharyngeal widths of upper airway and velum dimension with antero-superior repositioning of hyoid bone. Epworth sleepiness scale score improved significantly from baseline with clinically evident change in daytime discomforts. Significant decline in the mean apnoea/hypopnea index, oxygen desaturation index, respiratory disturbance index, heart rate, snoring and a significant increase in mean oxygen saturation of arterial blood was observed. No evident change noticed in occlusion except lower incisor inclination.

**Conclusion:**

The customized mandibular repositioning appliances are effective in the management of adult obstructive sleep apnoea with a significant improvement observed in the airway patency and polysomnography parameters with clinically non-significant effects on dental occlusion..

## INTRODUCTION

Obstructive sleep apnoea (OSA) is a potentially life-threatening disorder characterized by total or partial obstruction of the upper airway during sleep, leading to repetitive episodes of respiratory events such as apnoeas and hypopnoeas^1^. The cardinal manifestations of OSA are loud snoring, witnessed breathing pauses during sleep, excessive daytime sleepiness, and deficits in neurocognitive function adversely affecting the quality of life^2,3^. There has been an increasing awareness over the years that patients suffering from OSA are at risk from a wide range of medical complications as a result of nocturnal hypoxia they experience during sleep^1^.

In men older than 40 years of age, more than 50% snore occasionally, and about 10 to 15% snore regularly^4^. Among children and adolescents, the prevalence of primary snoring has been reported at 3.2% to 12.1%, with an estimated prevalence rate of 0.7% to 10.3% for OSA^5^. Decreased mandibular and maxillary length, skeletal retrognathism, high mandibular plane angle and inferiorly positioned hyoid bone are characteristic cephalometric findings in sleep-disordered breathing^6^.

The primary diagnosis comprises comprehensive history supported by the use of questionnaires such as Epworth sleepiness scale (ESS), detailed stomatognathic examination, body mass index (BMI), neck circumference calculation, and overnight PSG^7^. Lateral cephalograms are used for diagnostic purposes and to monitor the changes in airway in response to mandibular protrusion^6^.

Mandibular repositioning appliance (MRA) therapy is a unique, established approach that provides better sleep quality to patients suffering from snoring and mild-to-moderate OSA with excellent patient compliance^8^. Customized MRAs are designed to provide precision fit to act like a retainer for the purpose of eliminating any unwanted tooth movement^9^. MRAs were believed to exert their effects predominantly in the oropharynx and hypopharynx but some studies have suggested an effect on the retropalatal airway as well^10^. Only very few studies in literature explains about the amount of mandibular advancement with MRAs and possible side effects on occlusion^11^. Therefore, the current study aims at comparing the changes in pharyngeal airway space before and after treatment with a customized MRA, and to evaluate the nocturnal sleep patterns and daytime discomfort in patients with OSA along with the effects of MRA on occlusion.

## MATERIAL AND METHODS

The study protocol was approved by the registered ethics committee of the institution. All patients signed an informed consent form and agreed to participate in the study. The sample size consisted of 10 patients selected from 22 consecutive patients reported to the department of orthodontics suffering from snoring and disturbed sleep out of which 7 were males and 3 were females. These 10 patients were selected based on inclusion criteria and through analysing ESS questionnaire and remaining 12 patients were excluded from the study because of satisfying exclusion criteria. The duration of present study was fixed for a period of 2 years from the date of MRA delivery after registration in clinical trial registry (CTRI/2018/04/012923).

### Inclusion criteria

(1) Age range of 27.3-61.6 years; (2) patients diagnosed with OSA; (3) patients willing for MRA therapy; (4) sufficient number of teeth to retain the MRA.

### Exclusion criteria

(1) Maximum mandibular protrusion of less than 6mm; (2) medically compromised patients with severe systemic illness; (3) severe cariogenic status; (4) periodontally compromised dentition.

### Diagnosis

**Figure 1:**
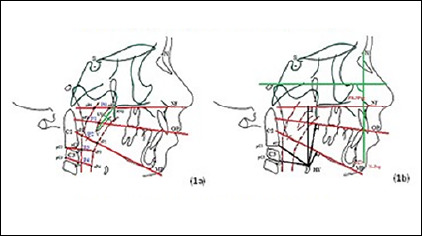
Cephalometric landmarks for reference planes, pharyngeal and velum dimension: A. Hyoid; B. Mandibular position. Definition of landmarks explained in Appendix I and II.

The primary diagnosis was established by the Epworth sleepiness scale (ESS), clinical examination and detailed history. The subjects in the study were assessed through subjective questionnaire to rule out any pre-existing systemic illness or under medication. The ESS score (range, 0-24) is usually elevated in sleep apnoea patients, indicating a propensity to fall asleep. An ESS score above 10 was considered abnormal^12,13^. All patients were instructed to fill the scale at the initiation of treatment. Subsequently the diagnosis was substantiated through assessment of the pharyngeal airway space on a lateral cephalogram and polysomnography (PSG). Standardized lateral cephalograms were taken at the first appointment and hard and soft tissue radiographic landmarks were located and tracings were made (Figure 1) (Appendix I and II)^7^. Among the subjects, facial form of 7 individuals were mesofacial and 3 individuals had a dolicofacial profile.

An overnight PSG recording with a complete PSG system (*Miniscreen Pro, Lowenstein medical technology, Hamburg, Germany*) was performed in a full fledge sleep lab. The Miniscreen Pro is a modern PSG system with up to 48 channels having simple integration of eight external signals and digital interface to prismaLINE therapy devices (*Weinmann, Lowenstein medical technology*). This level 1 PSG study comprised recordings of the pulse rate, apnoea/hypopnea index (AHI), oxygen desaturation index (ODI), respiratory disturbance index (RDI), arterial blood oxygen saturation (SaO_2_), respiratory movements, intensity and duration of snoring, and body positions. Monitoring SaO_2_ continuously with an ear probe, from which the minimum value of arterial blood oxygen saturation, expressed in percent (SaO_2_ nadir), was measured. The ODI was calculated and defined as the average number of episodes per hour when the oxygen saturation decreased more than or equal to 5% from the baseline value followed by determining AHI. According to the American Academy of Sleep Medicine (AASM), AHI is categorized into mild (5-15 events/hour), moderate (15-30 events/hour), and severe (>30 events/hour)^14^. AHI was originally calculated using previous AASM hypopnea scoring criteria (AHI(Chicago)), requiring either >50% airflow reduction or a lesser airflow reduction with associated >3% oxygen desaturation or arousal. AHIs using the “recommended” (AHI(Rec)) and the “alternative” (AHI(Alt)) hypopnea definitions of the AASM -Manual for Scoring of Sleep and Associated Events were then derived. For AHI(Rec), hypopneas were required to have > or =30% airflow reduction and > or =4% desaturation; and for AHI(Alt), hypopneas were required to have > or =50% airflow reduction and > or =3% desaturation or arousal^15^. RDI expresses the combined values of both AHI and respiratory effort related arousal (RERA).

## PROCEDURE

**Figure 2:**
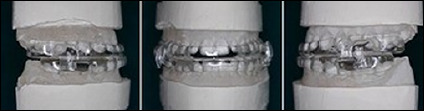
Customized mandibular repositioning appliance (MicrO2 Sleep & Snore device, ProsomnusTM, USA). Fabricated through CAD/CAM technology ensuring a precision fit.

**Figure 3:**
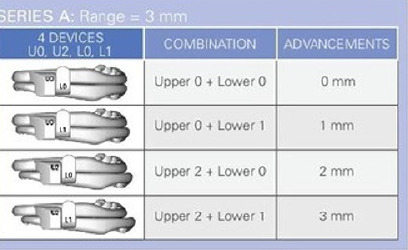
MicrO2 Sleep and snore device for mandibular sequential advancement using 4 set of appliance. Device number highlighted over the vertical post represents the amount of advancement.

A dental impression of the upper and lower arch was made using polyvinyl siloxane (PVS) impression material for the fabrication of MRA. Bite registration was carried out using George Gauge with mandibular advancement of 60% of maximum mandibular protrusion. Vertical opening of mandible was kept as low as possible around 3mm using standardized 3mm bite fork, which was custom made by *Prosomnus Sleep Technologies*, USA, in contrast to routinely available standard 2mm and 5mm bite fork. Custom made MRA (*MicrO_2_ Sleep & Snore device - Prosomnus^TM^ USA*) (Figure 2) was fabricated utilizing CAD/CAM digital technology. The appliance is made of high-grade polymethyl methacrylate (PMMA), which ensures decreased monomer leaching and further polymerization shrinkage. One-piece construction of appliance without moving parts assures precision fit to the teeth nullifying any unwanted tooth movement. Maintaining vertical opening of jaws at 3mm minimizes any temporomandibular joint (TMJ) complications. Mandibular advancement was accomplished using 4 sets of the appliance. The first set (namely U0, L0) of appliance was considered baseline in which the mandible is positioned 3mm distal to the initial recorded bite. The consecutive sets of appliances have 1mm of advancement each and by using the last set of appliance; the mandible will end at a point of 60% of maximum mandibular protrusion (Figure 3). In every 6 months, upgradation of consecutive set of appliance was car ried out and the patients were advised to use MRA every night during sleep for 2 years.

At the end of treatment lateral cephalograms, ESS and PSG were again repeated and the data were statistically evaluated. To determine the efficacy and efficiency of customized MRA, an elaborate examination of the pre and post-treatment dental occlusion was done on prepared study models in terms of overjet, overbite, intercanine and intermolar width in both the arches and interincisal angle, U1 - P P, L1 - MP as a lateral cephalometric parameter.

### Statistical analysis

Statistical analysis was performed using statistical package (SPSS software, IBM version 23, Armonk, New York, USA). The Kolmogorov-Smirnov test confirmed the sample normality. Cohen’s was used to calculate the effect size and it varied from 0.4 to 1.1. It was verified that all the measures were presented within the nullity hypothesis because all the values obtained indicated a value of *p*<0.05 and this also confirmed the use of parametric test. Statistical methods used were descriptive statistics including median, mean and standard deviation and inferential statistics including the students paired t test, which was used to compare pre-treatment and post-treatment cephalometric variables, ESS score, PSG parameters, and variables for detecting changes in occlusion. The level of significance was set at 0.05 at 95% confidence interval.

## RESULTS

The demographic data of the 10 consecutive patients reported to department of orthodontics with a complaint of snoring and disturbed sleep were analysed. The age of patients ranged from 27.3-61.6 years with a mean of 41.9 years. The subject’s height (cm) and weight (kg) were measured with a mean of 167.9±6.5 and 77.6±8.3, respectively. The BMI was calculated using the method described by Revicki and Israel (1986)^16^ (BMI = weight kg/height m^2^). Most of the subjects were overweight, with a BMI of 28.2kg/ m^2^ (range 25.3-32.6) and standard deviation of 2.57kg/m^2^. Post-treatment evaluation of BMI showed non-significant change from pre-treatment values and in the present study no correlation was established between pharyngeal airway width and BMI. The neck circumference ranged from 16-19.5 inches with a mean of 18.25±1.13 inches before treatment with clinically non- significant change observed after completion of treatment. All 10 patients successfully completed the duration of study with no exclusion of subjects at any point of time.

### Duration of appliance wear

The patients follow up was done every month and to evaluate the frequency of appliance wear the patients were asked to fill a feedback form describing the difficulties faced during appliance wear everyday using a series of 10, YES/NO questions along with record of hours of sleep per day for the respective month. The appliance was worn by the patients for a period of 2 years with a mean duration of 6.4 hours every night with a standard deviation of 0.516.

### Lateral cephalometric results

#### Pharyngeal dimensions

There was a significant increase in the mean pharyngeal widths of upper airway at P0, P1, P2, P3, P4 in relation to 5 reference planes NF, OP, MP, aC2-pC2, aC3-pC3, respectively, after MRA treatment (Table 1).

**Table 1:** Comparison of pre-treatment and post-treatment lateral cephalometric variables.

Variables		Mean	Standard deviation	t	Significance
3.2.1 Pharyngeal dimensions					
P0	Pre	18.0000	1.94365	-3.207	0.011
	Post	18.8000	1.81353		
P1	Pre	9.7000	2.21359	-5.014	0.001
	Post	11.5000	1.58114		
P2	Pre	10.6000	2.01108	-5.667	0.000
	Post	12.3000	1.88856		
P3	Pre	11.7000	1.94651	-5.667	0.000
	Post	13.4000	1.50555		
P4	Pre	12.6000	1.64655	-3.498	0.007
	Post	13.7000	2.00278		
3.2.2 Velum dimensions					
PNS-V	Pre	43.6000	3.30656	4.129	0.003
	Post	41.2000	2.25093		
LV-UV	Pre	9.8000	1.39841	-1.861	0.096
	Post	10.3000	.94868		
3.2.3 Hyoid position					
HY-NF	Pre	70.1000	3.41402	8.820	0.000
	Post	67.9000	2.92309		
HY-OP	Pre	57.1000	4.06749	11.699	0.000
	Post	55.0000	3.97213		
HY-MP	Pre	28.2000	2.44040	6.273	0.000
	Post	25.9000	2.33095		
HY-aC2	Pre	46.2000	2.61619	-6.708	0.000
	Post	47.7000	2.86938		
HY-aC3	Pre	37.9000	2.51440	-7.236	0.000
	Post	39.5000	2.41523		
3.2.4 Mandibular position					
FH-NPog	Pre	87.9000	1.79196	-1.464	0.177
	Post	88.4000	1.50555		
N┴ Pog	Pre	-3.6000	2.17051	2.739	0.023
	Post	-2.6000	1.83787		

Mean pharyngeal width P0 at the level of NF before treatment was 18.00±1.94 which showed statistically highly significant (*p*-value<0.05) change in the mean pharyngeal width of 18.80±1.81 following treatment. Accordingly, statistically significant increase was observed in mean pharyngeal width P1 at the level of OP from 9.70±2.21 to 11.50±1.58. A highly significant improvement in pharyngeal width P2 was evident in lateral cephalogram following MRA treatment compared to baseline (10.60±2.01 vs. 12.30±1.88; *p=*0.00). Mean pharyngeal width P3 at the level of aC2-pC2 and P4 at the level of aC3-pC3 before treatment was 11.70±1.94 and 12.60±1.64, respectively, which showed statistically significant improvement in pharyngeal width by 13.40±1.50 and 13.70±2.00.

Amount of mandibular advancement was standardized as 60% of maximum mandibular protrusion in each individual. The average amount of advancement in 10 subjects were within a range of 5-7mm with a mean of 6.11±0.32 and no evidence obtained to correlate with amplification of airway and amount of advancement since all individuals had similar amount of increase in pharyngeal airway width irrespective of amount of advancement and age of the patient.

## VELUM DIMENSIONS

The present study observed mean velum length PNS-V was significantly reduced following treatment when compared with pre-treatment values (43.60±3.30 vs. 41.20±2.25; *p*=0.003). Non-significant increase was noted with the mean velum thickness LV-UV following treatment (Table 1).

### Hyoid position

Highly significant decrease in mean hyoid position HY in vertical relation to NF (70.10±3.41 vs. 67.90±2.92), OP (57.10±4.06 vs. 55.00±3.97) and MP (28.20±2.44 vs. 25.90±2.33) was evident in post-treatment lateral cephalogram suggestive of superior repositioning of hyoid bone. In sagittal plane, mean hyoid position HY-aC2 and HY-aC3 showed a statistically significant increase of (46.20±2.61 vs. 47.70±2.86) and (37.90±2.51 vs. 39.50±2.41), respectively, suggestive of anterior repositioning of hyoid bone (Table 1).

### Mandibular position

Non-significant increase in the mean mandibular position FH-NPog from 87.90±1.79 to 88.40±1.50 with a p value of 0.177 and a significant increase in the mean mandibular position N┴Pog from -3.60±2.17 to -2.60±1.83 suggestive of anterior repositioning of pogonion after MRA treatment (Table 1). There was no correlation established between rotation of mandibular plane with amount of advancement and facial profile from the results of present study.

### Polysomnography results

**Figure 4:**
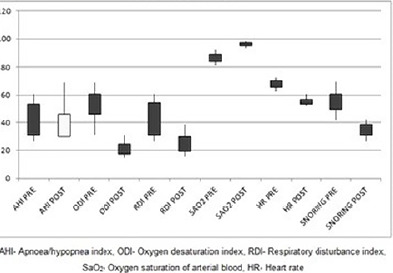
Graphical representation of pre-treatment and post-treatment comparison of mean diference in PSG parameters.

Overnight PSG studies revealed a highly significant reduction in the mean AHI, ODI, RDI, HR, snoring values and a significant increase in the mean SaO_2_ following MRA treatment (Table 2). A significant decrease in mean AHI following MRA treatment was observed (41.99±12.14 vs. 24.71±7.12) with similar findings among other parameters. Mean ODI and RDI values showed significant reduction (53.04±11.04 vs. 21.24±5.06) and (42.48±12.46 vs. 24.78±7.12), respectively. The mean heart rate among the study samples revealed a significant decrease from 68.03±3.07 to 55.00±8.38. On comparison with baseline findings, mean snoring rate showed a significant reduction from 55.19±8.38 to 34.16±4.66. A statistically significant improvement was evident in mean SaO_2_ levels following treatment (86.78±3.31 vs. 96.12±1.57; *p*=0.00) and overall findings were depicted through graphical representation (Figure 4).

**Table 2:** Comparison of pre-treatment and post-treatment polysomnography parameters.

Variables		Median	Mean	Standard deviation	t	Significance
AHI (/h)	Pre	40.45	41.9900	12.14985	7.625	0.000
	Post	23.3	24.7100	7.12436		
ODI (/h)	Pre	54.10	53.0400	11.04880	10.287	0.000
	Post	20.3	21.2400	5.06671		
RDI (/h)	Pre	40.85	42.4800	12.46478	7.518	0.000
	Post	23.6	24.7800	7.12270		
SaO2 (%)	Pre	86.55	86.7800	3.31186	-8.116	0.000
	Post	96.25	96.1200	1.57959		
HR (bpm)	Pre	68.8	68.0300	3.07790	13.598	0.000
	Post	53.95	55.0000	2.86201		
SNORING (%)	Pre	55.5	55.1900	8.38510	8.750	0.000
	Post	33.95	34.1600	4.66862		

AHI: Apnoea/hypopnea index; ODI: Oxygen desaturation index; RDI: Respiratory disturbance index; SaO2: Oxygen saturation of arterial blood; HR: Heart rate.

### Questionnaire based results

ESS questionnaire was used to evaluate subjective symptoms associated with OSA and to score daytime discomforts. On comparison with post-treatment findings, a statistically significant improvement in mean ESS score was observed following MRA treatment from 16.20±2.61 to 5.80±2.09 (Table 3).

**Table 3:** Comparison of pre-treatment and post-treatment ESS scor

Variables		Mean	Standard deviation	t	Significance
ESS	Pre	16.2000	2.61619	9.476	0.000
Score	Post	5.8000	2.09762		

### Effects on dental occlusion

The mean interincisal angle measured on pre and post-treatment lateral cephalogram exhibited a statistically significant decrease from 128.80±3.76 to 126.90±3.87 suggestive of proclination of incisors following MRA treatment. Mean U1 - PP and L1 - MP substantiates the above finding through highlighting non-significant decrease in U1 - PP from 111.60±2.11 to 111.40±2.17 and statistically significant increase in L1 - MP from 94.30±1.82 to 95.20±1.87 suggestive of proclined lower incisors (Table 4).

**Table 4:** Comparison of pre-treatment and post-treatment dental occlusion.

Variables			Mean	Standard deviation	t	Significance
Inter incisal angle (o)		Pre	128.8000	3.765	4.6696	0.0000
		Post	126.9000	3.871		
U1 - PP (o)		Pre	111.6000	2.118	1.0000	0.1717
		Post	111.4000	2.170		
L1 - MP (o)		Pre	94.3000	1.828	3.8571	0.0019
		Post	95.2000	1.873		
Inter canine width (mm)	Max	Pre	30.3000	0.918	1.4055	0.0967
		Post	30.1500	0.747		
	Mand	Pre	25.8500	0.883	0.0000	0.5000
		Post	25.9000	0.966		
Inter canine width (mm)	Max	Pre	38.3000	1.567	0.3611	0.3611
		Post	38.2500	1.703		
	Mand	Pre	34.1500	1.811	0.4803	0.3212
		Post	34.0500	1.535		
Overjet (mm)		Pre	3.4000	0.658	1.6269	0.0690
		Post	3.1000	0.774		
Overbite (mm)		Pre	2.6500	0.668	0.4285	0.3391
		Post	2.6000	0.459		

U1 - PP: Upper incisor to palatal plane; L1 - MP: Lower incisor to mandibular plane.

Mean intercanine width in the maxillary arch showed a non-significant change from 30.30±0.91 to 30.15±0.74 with a corresponding non-significant change evident in mandibular intercanine width (25.85±0.88 vs 25.90±0.96). Accordingly, mean intermolar width in maxillary and mandibular arch displayed a statistically non-significant decrease from 38.300±1.567 to 38.25±1.703 and 34.150±1.811 to 34.050±1.535, respectively. Mean overjet measured on pre and post-treatment study models showed statistically non-significant decrease from 3.400±0.658 to 3.100±0.774. Similarly, with overbite also non-significant change from 2.650±0.668 to 2.600±0.459 was noted (Table 4).

## DISCUSSION

MRAs has been used as an alternative treatment modality to CPAP for OSA treatment which is designed to improve upper airway dimension through alteration of jaw and tongue position, thereby preventing pharyngeal collapsibility^17^. MRAs were advocated as first-line therapy in patients with mild-to-moderate OSA and in more severe OSA patients who were intolerant to CPAP therapy by AASM^18^. In the current study, a positive effect was established in patients with severe OSA, a result that was supported by research of Cohen (1998)^19^, which reported that 9 out of 15 patients (60%) treated using MRA with moderate to severe OSA exhibited a post-treatment RDI of <5^19^.

MRAs can be either custom-made or bought ready-made over the counter. Ready-made MRAs offer the potential advantages of being easily available, at relatively low cost. Ready-made MRAs unfortunately are limited in their design, by the very fact that the manufacturer is attempting to cater to the needs of a very diverse population, with inherent differences in the size of their jaws and ability to protrude their mandible. In turn, the inherent malleability of these devices potentially compromises their fit^20^. In contrast to ready-made MRAs, customized devices are designed for a retainer-like fit, for the purpose of mitigating the risk of unwanted tooth movement through use of interdental embrasure adaption, or undercuts, for retention.

The current study substantiates the efficiency of custom fit MRA with very minimal negative effects in occlusion apart from angular relationships of incisors with their respective jaws. These devices provide a precision fit and sequential advancement through multiple set of appliance, which permit the patient gradually learn to advance the jaw forward, improving their effectiveness. Johal et al. (2017)^20^ compared the effectiveness of a custom-made versus ready-made MRAs in the management of OSA which demonstrated significant clinical effectiveness of a custom-made mandibular repositioning device over ready-made particularly in terms of patient compliance and tolerance. A comparative study by Vanderveken et al. (2013)^21^, concluded that excessive daytime sleepiness persisted in 45% and 55% of patients with the custom-made and ready-made MRAs, respectively.

The gold standard in diagnosing OSA is a full-night PSG recording in a laboratory with the primary outcome measure of apnoea-hypopnea index^22,23^. We found a strong positive correlation between PSG parameters of OSA severity and the amount of improvement in those PSG parameters with the device. In the current study, the mean oxygen saturation level improved from 86.78%±3.31% to 96.12%±1.57%. Similar improvements in oxygen saturation levels with MRAs were reported by Bernhold and Bondemark (1998)^24^, Bonham et al. (1998)^25^, Gavish et al. (2001)^26^, and Lowe et al. (1986)^27^. Mean AHI before treatment was 41.99±12.14, which in turn reduced to 24.71±7.12 after MRA treatment. This confirms the observation by Ferguson et al. (2001)^28^, who reported that 62.5% of their patients exhibited a reduction in the AHI by using an MRA.

Current study results shows mean ODI before treatment was 53.0400±11.04880 with a significant post-treatment change of 21.2400±5.06671. As per the study conducted by Fransson et al. (2001)^4^, 18 of the 22 OSA patients treated with MRA had a decreased ODI values by ≥50% with a significant change from 15.4 to 3.5 (*p*<0.001). Heart rate variability (HRV) reflects the status of the autonomic nervous system (ANS) in patients with physiological and pathological conditions, providing a unique index to identify OSA^29^. Numerous studies have demonstrated that the recurrence of the progressive-bradycardia/abrupttachycardia pattern observed in patients with OSA is likely the response of ANS to apnoeic events^30^. The mean heart rate among the study samples showed a significant decrease from 68.0300±3.07790 to 55.0000±8.38510. Gauthier et al. (2011)^31^ found that heart rate significantly decreased at follow-up (mean 40.9 months) compared to baseline after using an MRA. Similar findings were reported by Gotsopoulos et al. (2004)^32^, who assessed the heart rate derived from the 24-hour ambulatory blood pressure monitoring (ABPM).

ESS scores provide a measurement of a patient’s general level of daytime sleepiness, from low to very high in a variety of situations^33^. A positive correlation between pre-treatment values of ESS and AHI is helpful to distinguish patients with mild, moderate, and severe OSA according to the ESS scores. Murray (1991)^33^ reported similar findings and in our study, there was a highly significant reduction in the mean ESS score from 16.20±2.61 to 5.80±2.09 after MRA treatment. Improvement in ESS score with the MRA was also reported by Cozza et al. (2004)^34^, Hammond et al. (2007)^35^, Tan et al. (2002)^36^, Giannasi et al. (2013)^37^, and Blanco et al. (2005)^38^. As with any questionnaire-based scale, the ESS is also limited by the subject’s ability to read and understand the questionnaire and to answer the questions honestly.

The antero-posterior dimension at the smallest cross-section of the airway shows the severity of the obstruction of the upper airway in the sagittal plane. In the current study posterior airway space, i.e., the space between the base of the tongue and the posterior oropharynx in relation to NF, OP, MP, base of C2, the base of C3 was significantly increased from baseline with MRA therapy. Similar results were reported by Schmidt-Nowara et al. (1991)^39^ and another cephalometric study of 10 patients with OSA by Johnson et al. (1992)^40^ showed a 56% mean increase in posterior airway space when maximal mandibular protrusion was compared to the rest position after MRA treatment. The study sample showed a significant decrease in the mean velum length from 43.6±3.3 to 41.2±2.2 after MRA treatment. Similar results were reported by Eveloff et al. (1994)^41^ with an associated shortening of the soft palate length following the use of MRA.

The hyoid bone plays an important role in maintaining the upper airway dimension^42^. Because it serves as an anchor for the lingual musculature, it has received considerable attention, and inferior positions of this bone have been widely reported in patients with OSA^43^. Recent studies have suggested that the inferior position of the hyoid bone might not be a predisposing factor for airway obstruction, but rather a compensatory response^44^. Tourne (1991)^45^ noted that a drop in the hyoid position represents an attempt to secure a relatively constant anterior-posterior dimension of the airway. In the present study, before MRA treatment hyoid bone was found inferiorly and posteriorly placed in vertical relation to NF, O P, MP, and sagittal relation to aC2 and aC3, respectively, with a highly significant post-treatment change suggestive of hyoid bone repositioned superiorly and anteriorly. In a study conducted by Fransson et al. (2002)^7^ the average linear distances between the hyoid bone and the 2 reference lines, i.e., nasal line (NL) and mandibular line (ML), had increased significantly. The change in the position of the hyoid bone relative to the position of the mandible could be caused by a change in muscular activity – an adaptation to using the MPD for 6 to 7 hours per night – and not a permanent change in mandibular position, as in surgical mandibular advancement. The mandibular position in relation to the skull base was measured with an angular reading FH-NPog and a linear distance N┴Pog which showed a non-significant increase in mean FH-NPog and a significant increase in N┴Pog suggestive of anterior repositioning of the chin. As per the study conducted by Bondemark (2000)^46^, there was a significant change in mandibular position following MRA therapy and the observed change was temporary in nature.

### Limitations of the study

In the present study mode of investigation used to detect pharyngeal airway changes was lateral cephalogram. Cephalometric radiographs are 2-dimensional images and do not necessarily correlate with 3-dimensional measurements and also the imaging is usually performed in an upright posture (which is not a natural sleeping position compared with supine position)^7^. Also, the airway width was measured in the sagittal plane, which was not relevant according to Ryan et al. (1999)^10^ and Kyung et al. (2005)^47^, who observed that the oral appliance enlarges the pharynx to a greater degree in the lateral plane than in the sagittal plane at the retro palatal and retroglossal levels of the pharynx. Therefore, future studies could evaluate and compare different airway parameters, such as airway width in transverse plane and airway volume with CBCT after long-term use of MRA.

## CONCLUSION

The current study confirms that customized MRA is a valid therapeutic option for patients with mild-to-moderate OSA and for severe OSA patients who are non-compliant towards CPAP, if the improvement of respiratory parameters can be confirmed during follow-up PSGs. In this study, the patient’s reports of reduced daytime sleepiness, reduced snoring, and improvement in nocturnal sleep pattern, underline the therapeutic efficacy of customized MRA treatment, which is predominantly due to improvements in pharyngeal dimensions and hyoid bone position with minimal side effects in dental occlusion, even though angular relationship of incisors exhibited minor change in lateral cephalogram, which was clinically irrelevant.

## APPENDICES

### Appendix

**Appendix I.** Definition of cephalometric reference planes.

**Reference Planes**
NF - Nasal floor; a line connecting anterior nasal spine (ANS) and posterior nasal spine (PNS).
OP - Occlusal plane; a line connecting Mc and the incisal tip of the most prominent maxillary incisor (is).
MP - Mandibular plane; line tangent to the lower border of the body of the mandible through menton (Go-Me).
Base of C2 (2^nd^ cervical vertebrae) – a line connecting aC2 and pC2.
Base of C3 (3^rd^ cervical vertebrae) – a line connecting aC3 and pC3.

### Appendix

**Appendix II.** Definition of cephalometric variables measured in the study.

**Pharyngeal dimensions**
P0 - The linear distance between the posterior pharyngeal wall and posterior soft palate along NF (pP0-aP0) (mm);
P1 - The linear distance between the posterior pharyngeal wall and posterior soft palate along OP (pP1 - aP1) (mm);
P2 - The linear distance between posterior and anterior pharyngeal walls along MP (pP2 - aP2) (mm);
P3 - The linear distance between posterior and anterior pharyngeal walls along the base of C2 (pP3 - aP3) (mm);
P4 -The linear distance between posterior and anterior pharyngeal walls along the base of C3 (pP4 – aP4) (mm).

**Velum dimensions**
PNS - V Length of velum; distance between PNS and V (mm); LV- UV Thickness of velum; distance between LV and UV (mm).
Hyoid position
Hy-NF: Perpendicular distance from NF to Hy (mm); Hy-OP: Perpendicular distance from OP to Hy (mm); Hy-MP: Perpendicular distance from MP to Hy (mm);
Hy-aC2: Linear distance between Hy and aC2 (mm); Hy-aC3: Linear distance between Hy and aC3 (mm).
**Mandibular position**
***Angular measurement***
Facial angle: Angle formed by FH- NPog.

Linear measurement

N ┴ Pog: Horizontal distance between a perpendicular line from nasion to Pog.
